# On the Significance of the ADNT1 Carrier in *Arabidopsis thaliana* under Waterlogging Conditions

**DOI:** 10.3390/biom13050731

**Published:** 2023-04-24

**Authors:** Roberto Neri-Silva, Rita de Cássia Monteiro-Batista, Paula da Fonseca-Pereira, Mateus Dias Nunes, Ana Luiza Viana-Silva, Tamara Palhares Ribeiro, Jorge L. Pérez-Díaz, David B. Medeiros, Wagner L. Araújo, Alisdair R. Fernie, Adriano Nunes-Nesi

**Affiliations:** 1 National Institute of Science and Technology on Plant Physiology under Stress Conditions, Departamento de Biologia Vegetal, Universidade Federal de Viçosa, Viçosa 36570-900, MG, Brazil; 2 Max-Planck-Institute of Molecular Plant Physiology, Am Mühlenberg 1, 14476 Potsdam-Golm, Germany

**Keywords:** hypoxia, ATP/AMP transporter, stress responses

## Abstract

Among the adenylate carriers identified in *Arabidopsis thaliana*, only the AMP/ATP transporter *ADNT1* shows increased expression in roots under waterlogging stress conditions. Here, we investigated the impact of a reduced expression of *ADNT1* in *A. thaliana* plants submitted to waterlogging conditions. For this purpose, an *adnt1* T-DNA mutant and two *ADNT1* antisense lines were evaluated. Following waterlogging, *ADNT1* deficiency resulted in a reduced maximum quantum yield of PSII electron transport (significantly for *adnt1* and antisense Line 10), indicating a higher impact caused by the stress in the mutants. In addition, *ADNT1* deficient lines showed higher levels of AMP in roots under nonstress condition. This result indicates that the downregulation of *ADNT1* impacts the levels of adenylates. *ADNT1*-deficient plants exhibited a differential expression pattern of hypoxia-related genes with an increase in non-fermenting-related-kinase 1 (*SnRK1*) expression and upregulation of adenylate kinase (*ADK*) under stress and non-stress conditions. Together, these results indicated that the lower expression of *ADNT1* is associated with an early “hypoxic status” due to the perturbation of the adenylate pool caused by reduced AMP import by mitochondria. This perturbation, which is sensed by SnRK1, results in a metabolic reprogramming associated with early induction of the fermentative pathway in *ADNT1* deficient plants.

## 1. Introduction

Among all compounds that are transported between organelles, adenosine triphosphate (ATP) is perhaps the most important, as it is involved in the majority of the biochemical pathways of the cell [1]. Bearing in mind that in plants, ATP is synthesized mainly in the mitochondrial and chloroplast electron transport chains, ATP carriers are necessary to guarantee the supply of energy for the metabolic reactions occurring in the cytosol and other organelles [2]. Among the inner mitochondrial membrane carriers which are responsible for adenylate transport in Arabidopsis, the ADP/ATP carriers, named AAC carriers (AAC1, AAC2, and AAC3), represent the most abundant ones [3]. Despite being less abundant than AACs in the mitochondrial inner membrane, the ADNT1 (At4g01100) carrier is particularly interesting because, different from the AACs, it exports ATP in exchange for AMP, preferentially to ADP [4].

In Arabidopsis, *ADNT1* expression is mainly observed in heterotrophic organs with a high demand for ATP exported from mitochondria, such as seedlings, flowers, and roots [4]. Additionally, *ADNT1* expression is relatively high in senescent leaves [4,5]. However, both the physiological role and the metabolic context in which ADNT1 acts remain to be precisely elucidated. Considering the preference of ADNT1 carrier for cytosolic AMP [4], it is expected that ADNT1 plays a role under situations in which there is an elevation in the levels of cytosolic AMP. During hypoxic conditions, adenylate ratios are disrupted due to a decrease in mitochondrial electron transport chain (mETC) activity caused by oxygen limitation [6,7]. In this situation, the ATP produced by glycolysis becomes of utmost importance for plant survival [8]. Due to the lower production of mitochondrial ATP by the mETC, it is expected that less ATP is used to be converted to ADP by adenylate kinase (ADK) [9], which consequently results in accumulation of AMP in the cytosol. Bearing this in mind, the impacts of the reduced expression of *ADNT1* during hypoxia, a condition in which the flow of electrons through the mitochondrial electron transport chain is reduced, was investigated [8,10].

Under hypoxia, compelling evidence indicates that higher levels of AMP occur in the cytosol [11,12]. This situation is expected to favor the reaction catalyzed by the intermembrane space isoform of adenylate kinase (ADK), which synthesizes two ADP molecules using AMP and ATP as substrates [13]. These ADPs would re-enter the mitochondrial matrix via AAC carriers and then support the synthesis of ATP through oxidative phosphorylation [13]. In this context, a higher activity of ADNT1 would be required to export the ATP synthesized in the mETC in exchange for cytosolic AMP. Thus, given the transport preference of ADNT1 by AMP, it is likely that plants with reduced expression of this transporter are more sensitive to hypoxia. In such circumstances, the reduced import of cytosolic AMP by mitochondria, expected to occur in *ADNT1* deficient plants, might result in an elevated AMP/ATP ratio in the cytosol, which activates the energy sensor sucrose non-fermenting-related-kinase 1 (SnRK1) and the subsequent signaling response [14]. Thus, the role of ADNT1 carrier in *A. thaliana* plants submitted to waterlogging conditions were investigated by assessing physiological and metabolite changes along the stress period in both roots and leaves tissues. Additionally, the consequence of *ADNT1* downregulation on the expression of genes encoding proteins related to energy status in roots of plants under waterlogging stress was investigated. We further discuss a mechanism by which the perturbation in the adenylate pool, which is sensed by SnRK1, results in metabolic changes associated with the premature induction of the fermentative pathway in *ADNT1* deficient plants.

## 2. Materials and Methods

### 2.1. Plant Material and Growth Conditions

*Arabidopsis thaliana* seeds from wild-type (WT; ecotype Columbia-0), *ADNT1* T-DNA mutant (GABI-Kat 451B06), and two previously described *adnt1* antisense lines [4,5] were used in all experiments. Seeds were germinated on half-strength MS [15] (medium plates, pH 5,7, supplemented with sucrose 1% (*w*/*v*) in a growth chamber (150 μmol photons m^−2^ s^−1^ white light, 21 °C) under short-day conditions (8 h of light/16 h of dark). After 10 days, the seedlings were transferred to a commercial substrate (Carolina Soil, Kingston, NC, USA) and kept in a growth chamber under the same conditions previously mentioned.

### 2.2. Waterlogging Stress Conditions

The root system of 4-week-old plants was submitted to hypoxia by waterlogging, submerging the pots in trays with deionized water treated with N_2_ gas to eliminate the dissolved O_2_, forming approximately 0.2 cm of water blade above the substrate. During the experiment, samples from leaves and roots were collected in the middle of the light period, snap-frozen in liquid nitrogen, and stored at −80 °C until analyses. The samplings were performed before (time 0) and 3, 7, and 12 days after the onset of the waterlogging imposition.

### 2.3. Chlorophyll Fluorescence Measurements

The chlorophyll fluorescence was measured on fully expanded leaves of 4-week-old plants submitted to waterlogging conditions with the miniaturized pulse-amplitude-modulated photosynthesis yield analyzer (Mini-PAM) of H. Walz (Effeltrich, Germany) with the leaf clip holder for small size plants. After the dark adaptation of plants, the minimum fluorescence at open PSII centers in the dark-adapted state (*F_o_*) was determined. A saturating pulse of white light (800 ms, 3000 µmol photons m^−2^ s^−1^) was applied to determine the maximum fluorescence at closed PSII centers in the dark-adapted state (*F_m_*), and during actinic light illumination (*F_m_*′) [16]. The ratio of variable fluorescence to maximal fluorescence (*F*_v_/*F*_m_)—representing the potential quantum yield of PSII photochemistry—was measured in dark-adapted leaf tissue.

### 2.4. Metabolite Measurements

Frozen samples (50 mg), harvested in the middle of the light period, were homogenized with a mixture of methanol-chloroform-water (1:1:2.5), without Ribitol [17] for metabolites extraction. The methanol soluble phase was transferred to a 1.5 mL tube for the quantification of sugars, organic acids, and amino acids. The resulting pellet was subjected to three washes with the same extracting solution. Starch and total protein concentrations were quantified in the obtained pellet [18,19]. The supernatants and pellets were stored at −20 °C until further analyses. 

The contents of starch and soluble sugars (glucose, fructose, and sucrose) were analyzed as previously described [18]. The concentrations of total proteins and amino acids were quantified as described by Cross et al. (2006) [19]. The concentrations of malate and fumarate were determined as described by Nunes-Nesi et al. (2007) [20]. Chlorophyll a and b were determined before adding the mixture of chloroform-water. The absorbance readings were taken from each sample at 645 and 665 nm, as previously described by Porra et al. (1989) [21]. All measurements were performed in a VersaMaxTM Microplate Reader (Molecular Devices^®^).

### 2.5. Metabolite Profiling

Aliquots of 50–60 mg frozen leaf and root material were extracted with a mixture of chloroform–methanol–water for analyses of sugars, organic acids, and amino acids by gas chromatography-mass spectrometry (GC–MS) [22]. Peak integration was evaluated using TAGFINDER 4.0 software [23]. The mass spectra were cross-referenced with those from Golm Metabolome Database [24]. The amount of each metabolite was determined as the relative metabolite abundance, calculated by normalization of signal to that of Ribitol (internal standard) as described by Lisec et al. (2006) [22]. The data were calculated based on the fresh weight of leaves and roots.

### 2.6. Expression Analysis by qRT-PCR 

Total RNA was isolated from root material using TRIzol reagent (Ambion, Life Technology, Carlsbad, CA, USA) according to the manufacturer’s recommendations. Total RNA was treated with DNase I (DNase I Rnase Free; Celco, Ontario, CA, USA). The integrity of the RNA was checked on 1% (*w*/*v*) agarose gel, and the concentration was measured using a Nanodrop spectrophotometer. Finally, 500 ng of total RNA were reverse transcribed with a High-Capacity cDNA Reverse Transcription Kit (Thermo Fischer, Waltham, MA, USA) according to the manufacturer’s recommendations. Real-time PCR was performed on a MicroAmp™ Optical 96-Well Reaction Plate (Applied Biosystems, Waltham, MA, USA) using Ludwig SYBR Green qPCR Mix (Biotec, Los Angeles, SD, USA) according to the manufacturer’s recommendations. The primers used here were designed using the open-source program QuantPrime-qPCR primer design tool [25] and are described in Table S1. Relative transcript levels were calculated by relative quantification (standard curve method) and normalized using two constitutively expressed genes ACTIN2 (AT2G37620) and EF1a (AT5G60390). Three biological replicates were processed for each experimental condition.

### 2.7. Measurements of Adenylates Levels

ATP, ADP, and AMP were extracted using the trichloroacetic acid (TCA) method with minor modifications [26]. The pH of the extract was adjusted to 6.5 with 2.5 M KOH 0.5 M triethanolamine (TEA). The measurements were quantified using an ATP/ADP/AMP Assay Kit (Cat #: A-125; Biomedical Research Service Center, University at Buffalo, State University of New York). The luciferase bioluminescence was measured using a luminometer Perkin Elmer VICTOR^TM^ X5. Measurements were compared with a standard curve of ATP concentrations. Concentrations were calculated according to the manufacturer’s instruction. 

### 2.8. Experimental Design and Statistical Analysis

The experiments consisted of four genotypes (WT, *adnt1*, Line 10, and Line 22) submitted to waterlogging in a short time exposure with five time points (0, 12, 24, 48, and 72 h) and an extended one with four time points (0, 3, 7, and 12 days). Six biological replicates of each genotype were used for biochemical analyses, ten replicates for fluorescence analyses, and three replicates were used for RT-PCR analyses. The *t*-test was performed using the algorithm incorporated in Microsoft Excel (Microsoft Corporation, Seattle, WA, USA). Uneven variances were assumed and considered in the calculations. Values with *p* < 0.05 with the *t*-test were considered significant.

## 3. Results

### 3.1. The Expression of Mitochondrial Adenylates Carriers during Waterlogging Stress

**Figure 1:**
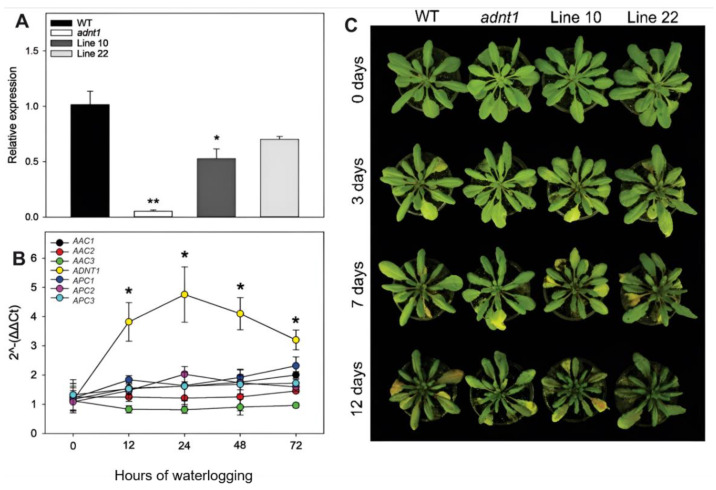
Phenotypic characterization of four-week-old Arabidopsis plants with reduced expression of *ADNT1* under root waterlogging treatment for 0, 3, 7, and 12 days (**A**). Expression analysis of *ADNT1* by quantitative real-time reverse transcription (RT)-PCR in leaves of the *Arabidopsis thaliana* wild-type (Col-0; WT), homozygous mutants (*adnt1*), and antisense lines (lines 10 and 22) (**B**). Changes in transcript levels of mitochondrial adenylates carriers in roots of four-week-old Arabidopsis (Col-0) plants during root waterlogging treatment for 0, 12, 24, 48, and 72 h. (**C**). Values are means ± standard error of three independent samples. Asterisks indicate values that were determined to be significantly different (*p* < 0.05) from the respective WT following the performance of the Student’s *t*-test. Two asterisks indicate values that were determined to be significantly different (*p* < 0.01) from the respective WT following the performance of the Student’s *t*-test.

To evaluate the level of *ADNT1* expression in the different lines used in all experiments, a RT-PCR analysis was performed in the leaves of four-week-old plants (Figure 1A). As expected, the *adnt1* mutant line exhibited a stronger reduction in *ADNT1* expression, followed by Line 10, which showed a reduction of 48%. Line 22 exhibited a mild and not significant reduction of ~30% in the expression of *ADNT1*. 

We next decided to evaluate the importance and response of adenylate carriers in roots from plants undergoing waterlogging stress. For that, the gene expression of seven mitochondrial carriers were analyzed in the roots of wild-type plants (Figure 1B). Interestingly, *ADNT1* was the only gene that displayed altered expression levels under this condition. The expression of *ADNT1* was higher at all time points with a peak 24 h after the onset of waterlogging stress in comparison with the other carriers.

### 3.2. Phenotypic Characterization of ADNT1 Deficient Plants during Waterlogging

Under optimal growth conditions for Arabidopsis, the phenotype of plants with reduced expression of *ADNT1* was similar to that exhibited by the wild-type, with no abnormal visible phenotypes in the mutants during the vegetative growth (Figure 1C), as previously observed [4,5]. After four weeks of cultivation, the plants were transferred to waterlogging conditions. Under this condition, plants from all genotypes exhibited mild chlorosis after seven days of oxygen deficiency (Figure 1C). After 12 days of waterlogging, visible stress symptoms were observed in all plants (Figure 1C). Genotypes deficient in the *ADNT1* expression exhibited the presence of purplish leaves to a slightly greater extent in comparison to the wild-type. In terms of rosette area and total leaf area, plants from all genotypes displayed a minor reduction from zero to 12 days under stress conditions; however, the extent of this phenotype was invariant across genotypes (Figure S1A,B).

We next evaluated the possible damages caused by both the low expression of *ADNT1* and root waterlogging to the photosynthetic apparatus in leaves. Plants deficient in the expression of *ADNT1*, particular *adnt1* and Line 10, displayed lower *F*_v_/*F*_m_ values at 7 (significant for *adnt1*) and 12 days (significant for *adnt1* and line 10) in comparison to the wild-type (Figure S2A). The content of chlorophyll *a* (Figure S2B), *b* (Figure S2C), as well as chlorophyll *a*/*b* ratio (Figure S2D), and total chlorophyll (Figure S2E) were also measured during the period of stress. The values of chlorophyll *a*, chlorophyll *a*/*b* ratio, and total chlorophyll decreased over time. The levels of chlorophyll *b*, on the other hand, remained stable during the period of root waterlogging. However, no statistical differences were observed between genotypes as compared to wild-type in any of the mentioned parameters.

### 3.3. Changes in Primary Metabolites in Leaves of Plants Deficient in the Expression of ADNT1 under Root Waterlogging Stress

**Figure 2:**
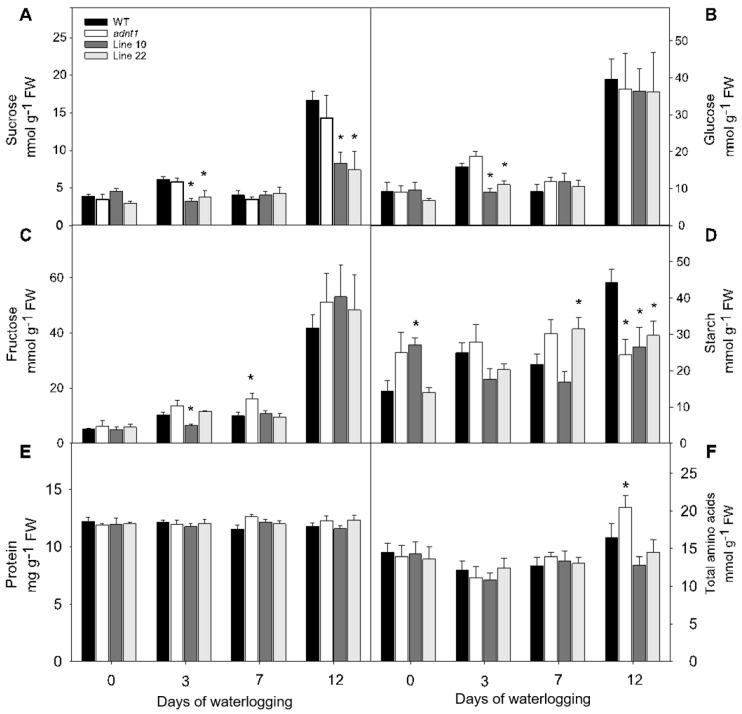
Variation of the main carbon and nitrogen-related compounds in leaves of four-week-old Arabidopsis plants with reduced expression of *ADNT1* under root waterlogging treatment for 0, 3, 7, and 12 days. Levels of sucrose (**A**), glucose (**B**), fructose (**C**), starch (**D**), protein (**E**), and total amino acids (**F**). Values are means ± standard error of six independent samples. Asterisks indicate values that were determined to be significantly different (*p* < 0.05) from the respective WT following the performance of the Student’s *t*-test.

For a more detailed characterization of the function of the ADNT1 carrier, biochemical analyses were first performed in leaves. Regarding the levels of soluble carbohydrates (sucrose, glucose, and fructose) in the middle of the light period, an increase was observed in all genotypes on day 7 after the stress period (Figure 2). The sucrose levels were reduced in leaves from the antisense lines on the third and on the twelfth day of stress (Figure 2A). Similarly, three days after the onset of the stress, *ADNT1* antisense lines exhibited lower glucose content compared to the wild-type (Figure 2B). For fructose, lower levels were observed at day 3 for Line 10 and significantly higher levels were found at day 7 for *adnt1* genotype. Therefore, no consistent changes were observed between the genotypes during the stress period for fructose (Figure 2C). Regarding the starch levels detected at day 0, a significant increase was observed for Line 10 in comparison to the wild-type (Figure 2D). We observed variation in starch levels without a clear correlation with the *ADNT1* expression pattern of the lines. Higher levels of starch were quantified in Line 22 at day 7, while all three *ADNT1* deficient lines showed reduced starch levels as compared to the wild-type at day 10 (Figure 2D).

Additionally, to verify the influence of *ADNT1* deficiency on nitrogen metabolism in plants under waterlogging conditions, the levels of protein (Figure 2E) and amino acids (Figure 2F) were evaluated. Both total free amino acids and soluble protein contents remained stable throughout the stress period, except for the twelfth day of stress, in which the *adnt1* mutant exhibited a significant increase in the total amino acid content.

### 3.4. Deficiency of ADNT1 Leads to a Differential Metabolic Response Following Waterlogging Conditions

To obtain a deeper view of the metabolic changes during waterlogging stress, we performed a metabolite profiling analysis in leaves and roots. A total of 35 metabolites were identified in leaves and 30 metabolites in roots.

**Figure 3:**
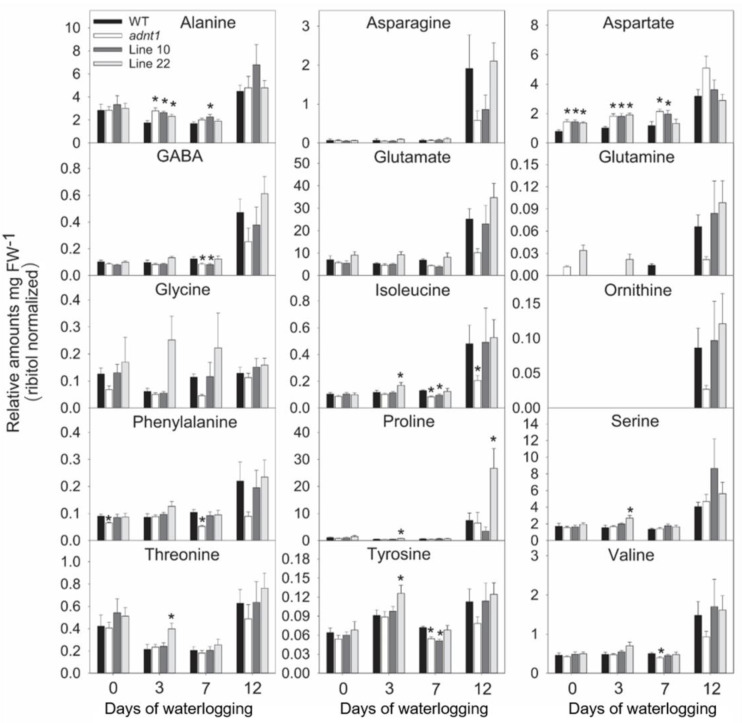
Amino acids from leaves of four-week-old Arabidopsis plants with reduced expression of *ADNT1* under root waterlogging treatment for 0, 3, 7, and 12 days. The data were normalized by fresh weight (FW), and internal control (Ribitol). Values are means ± standard error of six independent samples. Asterisks indicate values that were determined to be significantly different (*p* < 0.05) from the respective wild-type (WT) following the performance of the Student’s *t*-test.

**Figure 4:**
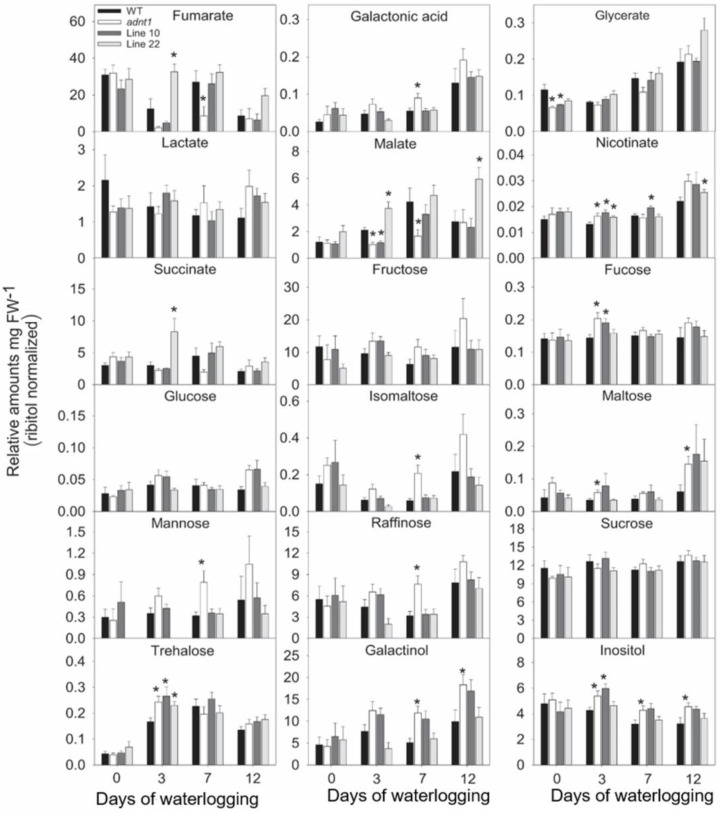
Organic acids, sugars, and alcohols sugar from leaves of four-week-old Arabidopsis plants with reduced expression of *ADNT1* under root waterlogging treatment for 0, 3, 7, and 12 days. The data were normalized by fresh weight (FW), and internal control (Ribitol). Values are means ± standard error of six independent samples. Asterisks indicate values that were determined to be significantly different (*p* < 0.05) from the respective wild-type (WT) following the performance of the Student’s *t*-test.

**Figure 5:**
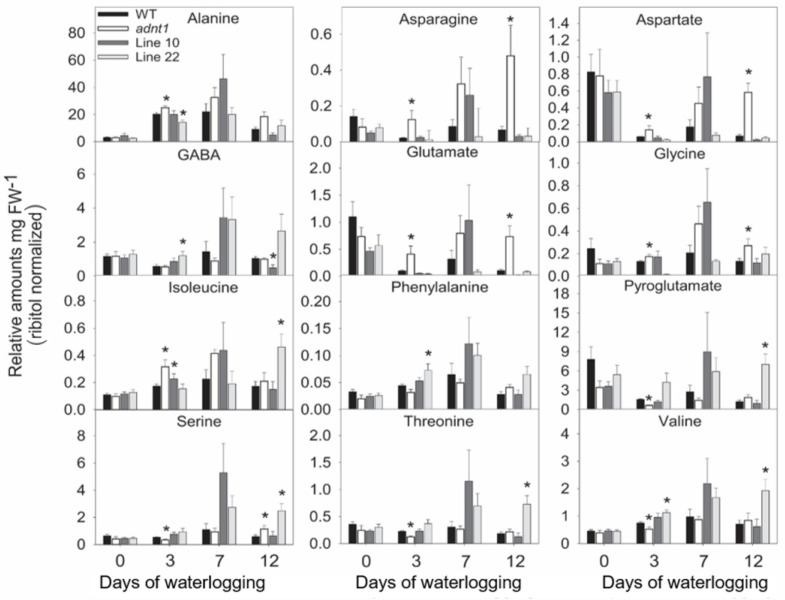
Amino acids levels in roots of four-week-old Arabidopsis plants with reduced expression of *ADNT1* under root waterlogging treatment for 0, 3, 7, and 12 days. The data were normalized by fresh weight (FW), and internal control (Ribitol). Values are means ± standard error of six independent samples. Asterisks indicate values that were determined to be significantly different (*p* < 0.05) from the respective wild-type (WT) following the performance of the Student’s *t*-test.

In leaves (Figure 3 and Figure 4), relatively few differences were found between the genotypes at time 0. Among these differences, higher levels of aspartate were found in *ADNT1* deficient lines compared with the wild-type in leaves (Figure 3). Reduced levels of glycerate were also observed in *adnt1* mutants and Line 10 at day 0 in roots (Figure 4). On the third day of stress, a greater number of changes was verified in the levels of metabolites from *ADNT1* mutant lines in comparison to the respective wild-type leaves. In general, a decrease in malate level in roots (significantly for *adnt1* mutant, Line 10 and Line 22; Figure 4) was observed. At the same time, there was an increase in key metabolites related to stress responses, such as alanine (significantly for *adnt1* at day 3), aspartate (significantly for *adnt1* at days 3 and 10), nicotinate (significantly for all mutants at day 3), trehalose (significantly for all mutants at day 3), and inositol (significantly for *adnt1* at all time points and for line 10 at day 3) (Figure 4 and Figure 5). Additionally, the levels of some sugars were increased after three days of stress, including those of fucose and maltose, which were both significantly elevated at day 3 for *adnt1* and Line 10 and at day 12 for *adnt1* (Figure 4). From the seventh day under waterlogging, a decrease was observed in the levels of isoleucine, phenylalanine, valine, and GABA (Figure 5).

**Figure 6:**
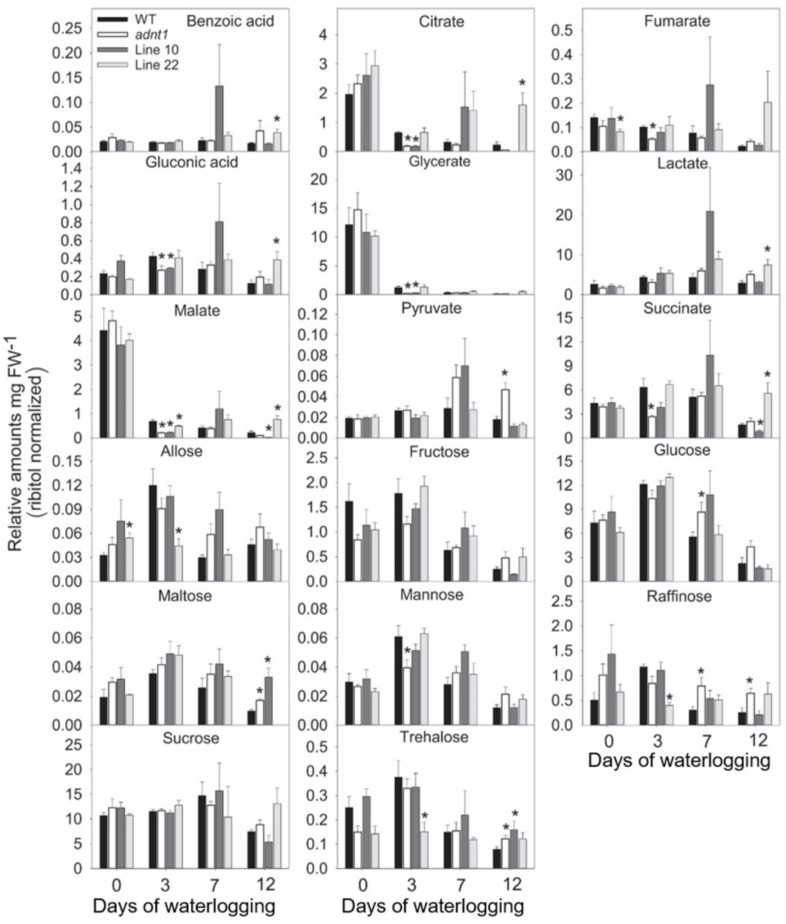
Organic acids, sugars, and sugar alcohols extracted from roots of four-week-old Arabidopsis plants with reduced expression of *ADNT1* under root waterlogging treatment for 0, 3, 7, and 12 days. The data were normalized by fresh weight (FW), and internal control (Ribitol). Values are means ± standard error of six independent samples. Asterisks indicate values that were determined to be significantly different (*p* < 0.05) from the respective wild-type (WT) following the performance of the Student’s *t*-test.

Later, at 12 days of root waterlogging, there were few differences between the genotypes, with *adnt1* mutants showing higher levels of maltose, galactinol, and inositol in their roots (Figure 6). Before the onset of the stress period, no significant alterations were observed in root metabolite levels in the *ADNT1* deficient lines. At day 3 of stress, we observed a significant increase in the levels of alanine, and isoleucine in *adnt1* mutant roots. Additionally, higher levels of asparagine, aspartate, glutamate, and glycine were observed only in the *adnt1* mutant compared to the respective wild-type (Figure 5). On the other hand, citrate, fumarate, gluconate, and glycerate exhibited a decrease in the *adnt1* mutant and Line 10 in comparison with the wild-type (Figure 6). At seven days of waterlogging, only glucose and raffinose showed a difference between the genotypes, with an increase in the *adnt1* mutant compared with the wild-type (Figure 6). On the twelfth day, the levels of maltose, asparagine, aspartate, glutamate, glycine, pyruvate, and raffinose were significantly higher only in *adnt1* mutant (Figure 6).

### 3.5. Expression of Genes Encoding Proteins Related to Energy Status in Roots of ADNT1 Deficient Plants under Waterlogging

**Figure 7:**
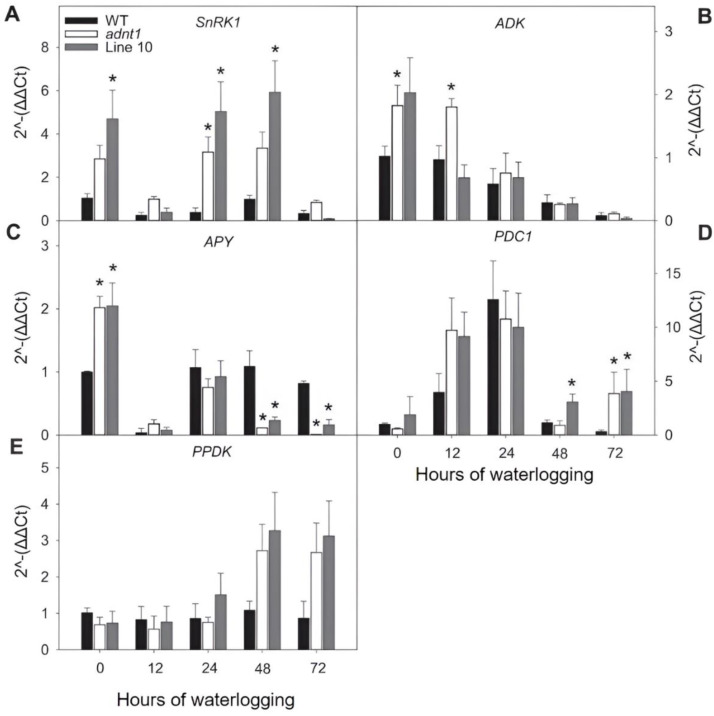
Changes in transcript levels in four-week-old, short-day-grown Arabidopsis plants with reduced expression of *ADNT1* under root waterlogging treatment for 0, 3, 7, and 12 days. Transcript abundance is shown for genes associated with the change in energy status of the cell SNF1-related kinase 1 (*SnRK1*; (**A**)), adenylate Kinase (*ADK*; (**B**)), apyrase (*APY*; (**C**)), pyruvate decarboxylase 1 (*PDC1*; (**D**)), and pyruvate phosphate dikinase (*PPDK*; (**E**)). Asterisks indicate values that were determined to be significantly different (*p* < 0.05) from the respective wild-type (WT) following the performance of the Student’s *t*-test.

To evaluate the impact of reduced expression of *ADNT1* in plants submitted to waterlogging conditions, the expression of genes related to energy status and AMP metabolism was evaluated (Figure 7). The gene selection for this analysis was based on signaling of energy deficiency associated with stress, *SnRK1*, modulation of adenylates pool, adenylate kinase (*ADK*), diphosphate kinase activity which produces AMP, apyrase (*APY*), and pyruvate phosphate dikinase (*PPDK*), and the gene marker for hypoxic stress, pyruvate decarboxylase (*PDC1*). Interestingly, the gene encoding *SnRK1* was significantly higher expressed in *ADNT1* deficient plants throughout the waterlogging period at the time points 0, 24, and 48 h of stress, with a significant increase at 24 h in the *adnt1* mutant, and 0, 24, and 48 h for Line 10 (Figure 7A). The expression of ADK was significantly higher only at 0 and 12 h in the *adnt1* mutant line (Figure 7B). The *APY* showed a pattern of reduction in expression during the analyzed stress period for all evaluated genotypes (Figure 7C). Moreover, both *adnt1* mutant and Line 10 exhibited higher expression of *APY* at time 0 and lower expression at 48 and 72 h in comparison with their wild-type counterparts. Line 10 also showed higher expression of *PDC1* at 48, while both *adnt1* mutants and Line 10 showed higher expression of *PDC1* at 72 h of root waterlogging in comparison with the wild-type (Figure 7D). No significant differences were observed in the expression of *PPDK* in comparison with the wild-type (Figure 7E).

### 3.6. Downregulation of ADNT1 Gene Impacts the Levels of AMP in Roots

**Figure 8:**
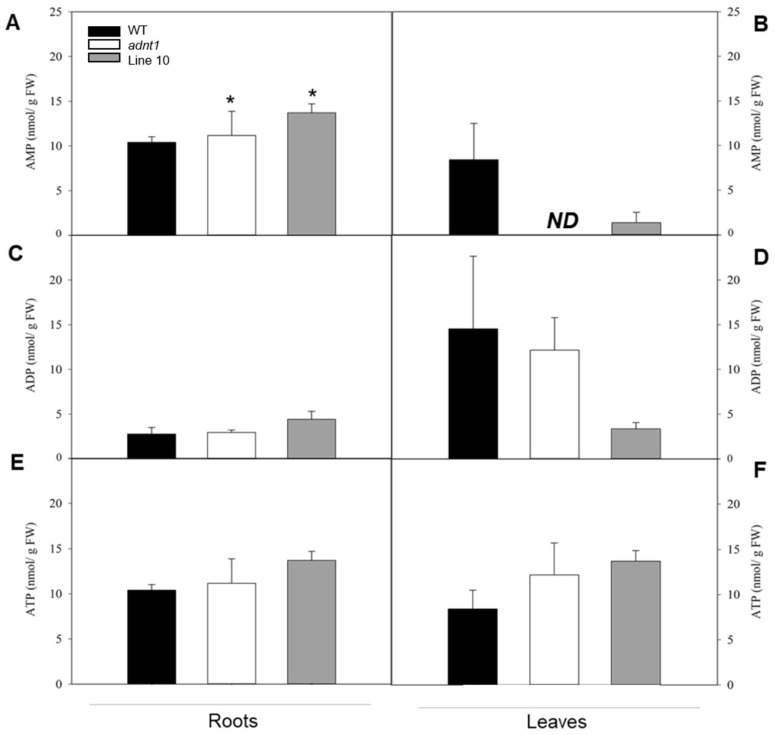
Adenylates levels in roots and leaves from 4-weeks-old wild-type (WT), *ADNT1* deficient lines in Arabidopsis plants. AMP (**A**,**B**), ADP (**C**,**D**), and ATP (**E**,**F**), Values (nmol/g FW) as means ± standard error of three independent samples. Independent sample *t*-test using Excel Statistics Software. Asterisks indicate values that were determined to be significantly different (*p* < 0.05) from the respective WT following the performance of the Student’s *t*-test. ND (Not detect)—Value below detection limit.

Aiming to evaluate adenylate levels in *ADNT1* deficient plants, we measured ATP, ADP, and AMP in roots and leaves from the wild-type and the mutants at the time point 0 (Figure 8). No significant changes were observed in the levels of ATP and ADP in both roots and leaves nor in the levels of AMP in leaves. Conversely, both *adnt1* mutants and Line 10 exhibited significant higher levels of AMP in roots in comparison to their respective wild-type plants. Despite the elevation in AMP, no significative changes were observed between *ADNT1* mutants and the wild-type for the ATP/AMP and ATP/ADP ratios (Figure S3).

## 4. Discussion

### 4.1. Deficiency of ADNT1 Expression Leads to an Early “Hypoxic Status” in A. thaliana Plants under Waterlogging

Oxygen-limited conditions primarily affect aerobic respiration, resulting in an energy deficit and ultimately in cell and tissue death [27]. Under this condition, changes in energy charge (ATP/ADP and ATP/AMP ratios) trigger metabolic modifications that ultimately result in an early senescence process [28]. Following waterlogging stress, *ADNT1*-deficient plants displayed rapid hypoxic symptoms as evidenced by both declines in the photochemical efficiency and lower starch content, at the end of the hypoxic treatment (Figure 2D). These results suggest that the deficiency of the *ADNT1* gene might result in an unbalanced ATP/ADP ratio in these plants in comparison to the wild-type. These results support the previous assumption that the ADNT1 carrier plays an alternative role in stress conditions such as hypoxia or carbon starvation [5,29] which deplete cellular ATP.

The increased expression of *SnRK1*, *ADK*, and *PDC1* in *ADNT1*-deficient plants (Figure 7) was followed by metabolic changes, such as alanine accumulation (Figure 3), which is one of the first responses of plants in conditions of low oxygen concentration [30]. The fact that *ADNT1*-deficient plants exhibited increased alanine levels in both leaves and roots than that found in the wild-type (Figure 3 and Figure 5) indicates that these plants achieve hypoxic status prematurely because the synthesis of alanine is an important strategy for carbon storage under flooding conditions [31,32] and for maintaining osmotic potential in stressed tissues [31]. All this added to the fact that aspartate, trehalose, and inositol, other metabolites usually accumulated during hypoxic stress [33], also accumulated at higher levels in the mutants, supports the hypothesis that *ADNT1* deficient plants are likely more sensitive to root waterlogging.

In plants under hypoxia, the mETC decreases its activity considerably [34]. As a result, the NADH/NAD^+^ ratio in the mitochondrial matrix increases and the TCA cycle slows down its activity leading to an accumulation of the TCA cycle intermediates [35]. In addition, the slowdown of the TCA cycle leads to a decrease in pyruvate metabolization in the cytosol, thus reducing substrate availability in the TCA cycle [36]. In agreement, *ADNT1*-deficient plants exhibited a slightly larger reduction in organic acids (citrate, fumarate, malate, and succinate) than that exhibited by the wild-type, particularly in roots, which are tissues directly exposed to hypoxic conditions. Because the mETC is inhibited under hypoxic conditions, the ATP production is considerably affected, making glycolysis the main pathway to supply energy metabolism [37]. Because of the lower capacity of glycolysis to produce ATP, larger sugar consumption may occur under hypoxia. However, low oxygen may also cause an increase in sugar content in the aerial part due to the impairment of sugar translocation to roots [38]. The last case is probably what occurred in *ADNT1*-deficient genotypes because leaves generally showed moderate increase in sugars/sugar alcohols (isomaltose, maltose, trehalose, galactinol, inositol) over time (Figure 4), while in roots there was a primary increase followed by a reduction for most detected sugars at the end of the stress period (Figure 6). That said, these results suggest an early response of *ADNT1*-deficient plants to hypoxic conditions. Because both *adnt1* and line 10 mutants exhibit higher levels of AMP in roots under nonstress condition (Figure 8), it can be concluded that the downregulation of *ADNT1* gene impacts the levels of adenylates in roots. It should be noted that the results, from both previous studies [4,5] and from the current one, do not point to a higher level of stress in the mutants under normal conditions. This can be assumed because (i) there were no differences in the photochemical efficiency among mutants and the wild-type at the time point 0 and (ii) the gene expression of the established senescence marker gene SAG12 did not change in the *ADNT1* mutants compared with the wild-type under nonstress condition [5]. It is also noteworthy that this result is in consonance with our hypothesis that absence of ADNT1 carrier generates an early “hypoxic status” due to perturbation of the adenylate pool caused by reduced AMP import by mitochondria, which might be a consequence of an earlier and higher unbalance in the ATP/AMP ratio. This would be anticipated to promote the stress responses prematurely in *ADNT1*-deficient plants, as can be seen by the increase of alanine levels, and other metabolic stress markers such as aspartate, trehalose, and inositol.

### 4.2. Reduced Expression of ADNT1 Affects the Expression of Genes Related to Waterlogging Stress Responses

SnRK1 is a key sensor of the cellular energy status which can be regulated by the levels of sugars and adenylates [39,40,41]. The expression of this gene is greater in *ADNT1* deficient plants in both stress and non-stress conditions (Figure 7A). This result suggests that the expression of *SnRK1* might be altered by the perturbation in the adenylate pool caused by *ADNT1* disruption and not directly by the waterlogging stress as already suggested [38,42]. Furthermore, *ADK* and *APY* exhibited higher expression under non-stressing conditions in *ADNT1* deficient lines. Considering that ADNT1 imports AMP (in exchange for mitochondrial ATP) from the cytosol to the mitochondria [4], it is expected that *ADNT1* deficient plants possess a greater AMP level in the cytosol. Accordingly, our data demonstrated that both *adnt1* mutants and Line 10 exhibit significant higher levels of AMP in roots in comparison to their respective wild-type plants under nonstress condition (Figure 8). Because of the higher level of AMP in *ADNT1*-deficient plants, the action of ADK is required to equilibrate the levels and ratios of adenylates [3,43], which is in consonance with the elevated expression of *ADK* in the *adnt1* mutant at 0 and 12 h of stress (Figure 7B). The highest expression of *APY* was observed under non-stress conditions in plants deficient in *ADNT1*. Apyrase produces AMP through ATP hydrolysis by diphosphate activity, which participates in adjusting the adenylate pool [35,44]. Furthermore, after 48 and 72 h of waterlogging stress, the expression of *APY* dropped in *ADNT1*-deficient plants (Figure 7C). Although the exact mechanism explaining the pattern of *APY* expression cannot be unequivocally elucidated here, it seems reasonable to suggest a possible explanation for this observation. The mETC itself is affected by oxygen deprivation, thus decreasing the production of ATP and the adenylate pool. A reduction in the adenylate pool caused by hypoxia would influence the activity of APY, which would have less ATP to hydrolyze, consequently leading to lower AMP in the cytosol. Additionally, the greater expression of *PDC1* period was observed in the *ADNT1* deficient plants only during the waterlogging stress (at the time point 72 h) (Figure 7D,E). This gene is strongly induced during hypoxia, in which PDC1 catalyzes the first step in the anaerobic fermentation pathway, converting pyruvate to acetaldehyde [45]. The higher expression of *PDC1* in *ADNT1* lower expression lines suggests an early and more expressive induction of the fermentative pathway in *ADNT1* deficient plants, in response to the drop of oxygen levels, which is followed by pyruvate accumulation during the stress.

## 5. Conclusions

Our findings indicate that the ADNT1 carrier performs a role in plants during waterlogging stress, being the only mitochondrial adenylate carrier upregulated in the roots of plants submitted to this stress. Future studies are clearly required to provide a better understanding of how ADNT1 and the other mitochondrial adenylate carriers coordinate the distribution of ATP, ADP, and AMP under stress. Hitherto, it can be assumed that the absence of ADNT1 carrier perturbates the adenylate pool in consequence of a reduced AMP import by mitochondria. This disturbance seems to be associated to the generation of an early “hypoxic status”. This perturbation in the adenylate pool, which is sensed by SnRK1, results in metabolic changes associated with the premature induction of the fermentative pathway in *ADNT1*-deficient plants. These early responses can result in rapid reserves consumption under prolonged stress. These consequences can be disadvantageous to plants and lead to a greater susceptibility to waterlogging conditions.

## Data Availability

Data is contained within the article or supplementary material.

## Supplementary Materials

The following supporting information can be downloaded at: https://www.mdpi.com/article/10.3390/biom13050731/s1, Figure S1: Phenotypic characterization of four-week-old Arabidopsis plants with reduced expression of ADNT1 carrier under root waterlogging treatment for 0 and 12 days, Figure S2: Photosynthetic pigments content and maximum quantum yield of PSII electron transport of four-week-old Arabidopsis plants with reduced expression of ADNT1 carrier under root waterlogging treatment for 0, 3, 7, and 12 days, Figure S3: ATP/ADP and ATP/AMP Ratio in roots (A and C) and leaves (B and D) of WT, adnt1 and Line 10 in Arabidopsis. Table S1: List of primers used for QuantPrime-qPCR analyses.
