# Supplementary material for: On the Significance of the ADNT1 Carrier in Arabidopsis thaliana under Waterlogging Conditions
Source: Biomolecules. 2023 Apr 24;13(5):731. doi: 10.3390/biom13050731 (PMC10216781; doi:10.3390/biom13050731)
Supplement: Supplementary file 1 [file biomolecules-13-00731-s001.zip › biomolecules-2315937-supplementary.pdf]

SUPPLEMENTAL MATERIAL

Table S1. List of primers used for QuantPrime-qPCR analyses.

Gene	ID	Forward	Reverse
Actin	AT3G18780.2	CTTGCACCAAGCAGCATGAA	CCGATCCAGACACTGTACTTCCTT
EF1a	AT5G60390.1	TTCGCTGTTAGGGACATGAGG	GTCGATCATAACGAAAGTCTCA
AAC1	AT3G08580.1	CTCCTCACTGGTGACTTACAGGAC	TTGTACTTGACAGCTTCACCAGAC
AAC2	AT5G13490.1	GGTTCGTTGTGGAGAGGAAACACC	ATGCAAAGTTCAAGGCCTGAGTGG
AAC3	AT4G28390.1	CGAAGGAATGCTTGCTCTTTGGAG	CGCAAAGTTTAAAGCCTGAGTGG
ADNT1	AT4G01100.1	GGGGCTTAGCCAAACTCTTT	TGAGAAAAGAGGAGCCCAAA
APC1	AT5G61810.1	GGAGCCAGGTCCTTTGATACAAC	TCAGAAACTCTTGGCCCATGC
APC2	AT5G51050.1	GAAGACAAAGCGGATATAGGCAC	TCGTGTAAGTCTGCAACCGAGTT
APC3	AT5G07320.1	CGAGATGGGCGTGTTGATTACC	AATGCCGGCCTTAACAAGAGC
SnRK1.2	AT3G01090.1	CAACCGAACCCAGAATGATGGC	AGGCACGGAAACGATTGTCCAG
ADK	AT2G37250.1	ATGGAGTTTGACTTACCTGGAGG	GTGAACTTATGCTGCGACAGAC
APY	AT3G04080.1	GGATTCGGATTGAAGCCATCAC	GCAAATACCCAAAATTGCCTTCCT
Pdci	AT4G33070.1	CGACCGTTGGGTTTAATCATGT	CCATGATAAAGCGTACATGGAAA
PPDK	AT4G15530.5	TGAGAAGGGTCATACCGTGAGC	ACTCCGCCTCTTTCGCAATCTC

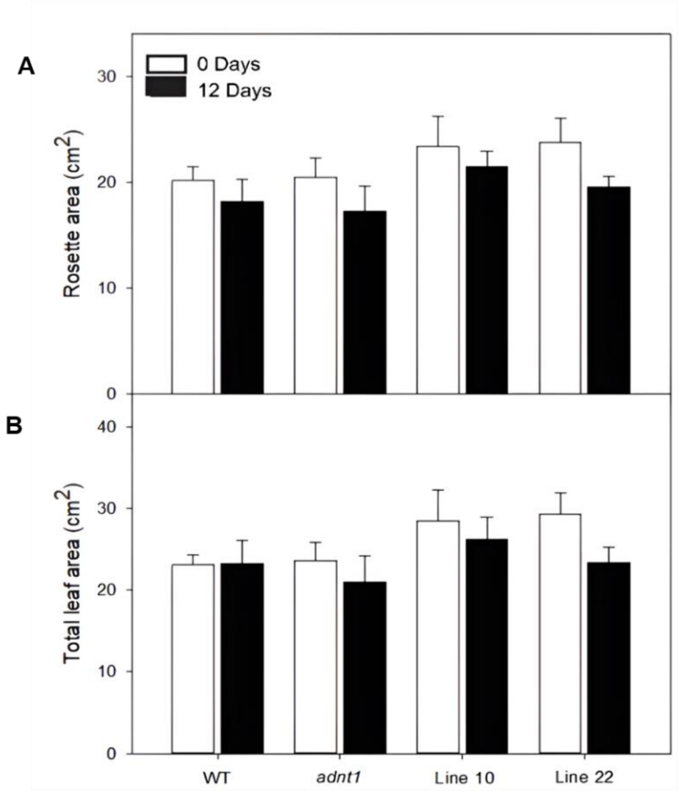

Figure S1. Phenotypic characterization of four-week-old Arabidopsis plants with reduced expression of ADNT1 carrier under root waterlogging treatment for 0 and 12 days. Rosette area (**A**) and total leaf area (**B**). Values are means ± standard error of six independent plants. Asterisks indicate values that were determined to be significantly different ($P < 0.05$) from the respective WT following the performance of the Student's *t*-test.

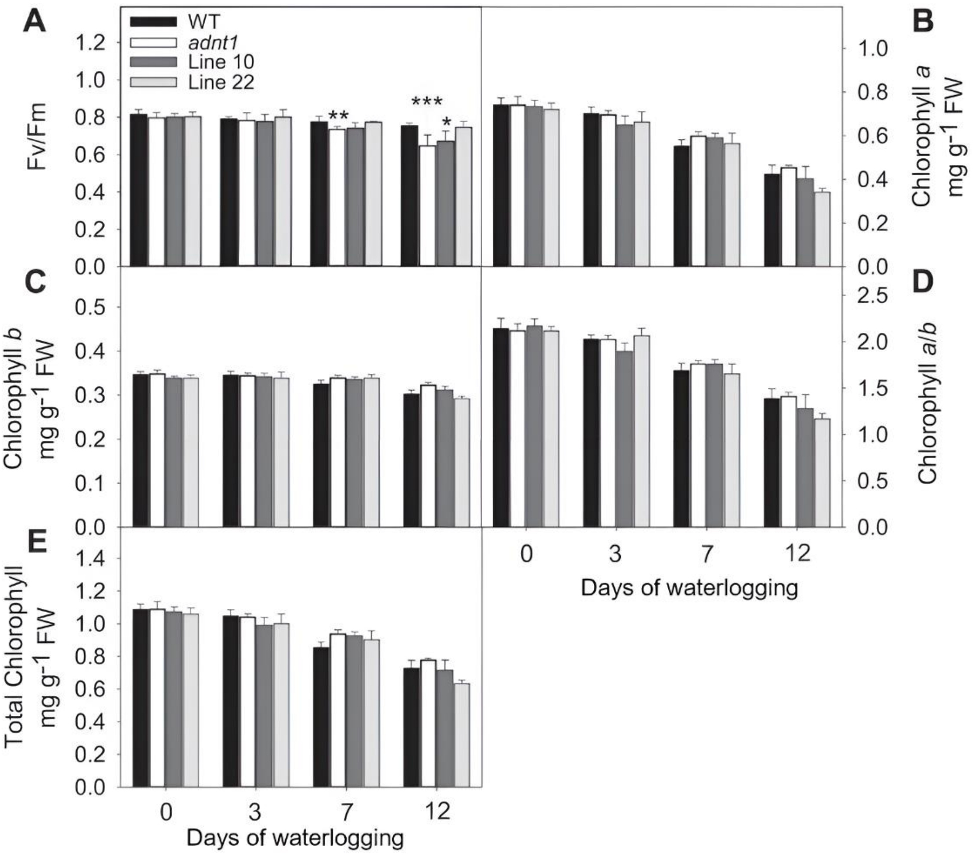

Figure S2. Photosynthetic pigments content and maximum quantum yield of PSII electron transport of four-week-old Arabidopsis plants with reduced expression of *ADNT1* carrier under root waterlogging treatment for 0, 3, 7, and 12 days. F_v/F_m (**A**), Chlorophyll *a* (**B**), Chlorophyll *b* (**C**), Chlorophyll *a*/*b* (**D**), and Total Chlorophyll (**E**). Values are means ± standard error of six independent samples to pigment analyses and ten independent samples to fluorescence analysis. Asterisks indicate values that were determined to be significantly different ($P < 0.05$) from the respective WT following the performance of Student's *t*-test.

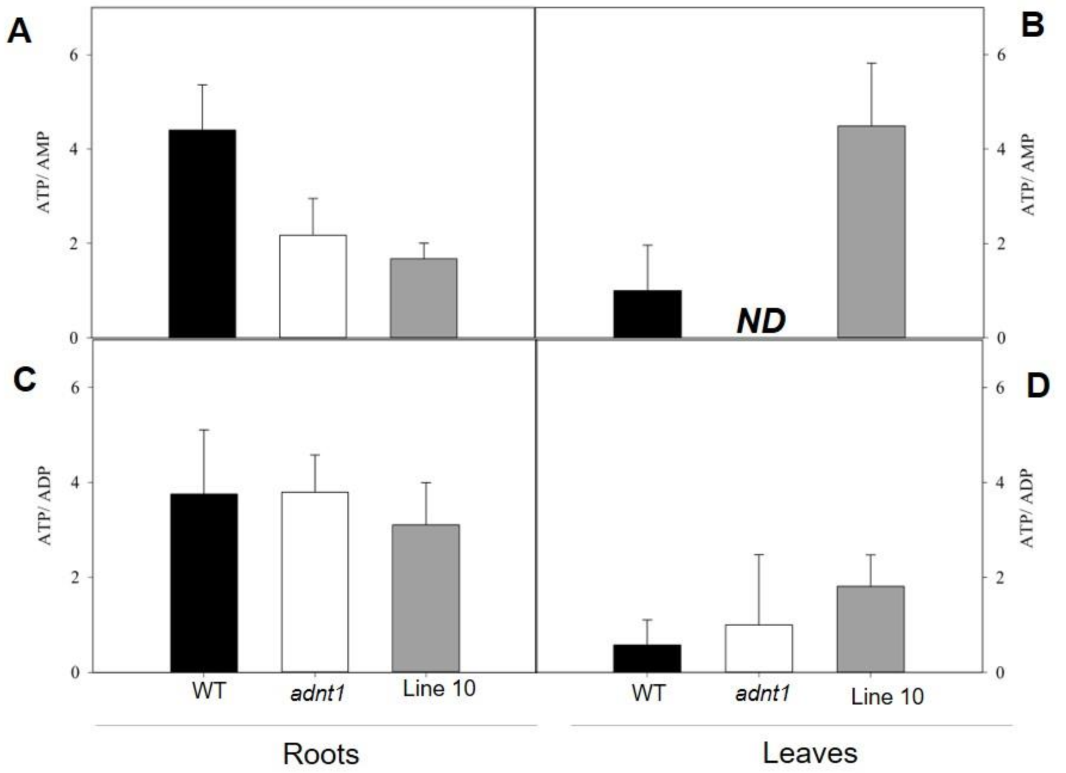

Figure S3. ATP/ADP and ATP/AMP Ratio in roots (A and C) and leaves (B and D) of WT, *adnt1* and Line 10 in Arabidopsis. ND (Not detect)- Value below detection limit. Asterisks indicate values that were determined to be significantly different (P < 0.05) from the respective WT following the performance of Student's *t*-test.
